# Recumbent Stepper Submaximal Test response is reliable in adults with and without stroke

**DOI:** 10.1371/journal.pone.0172294

**Published:** 2017-02-16

**Authors:** David R. Wilson, Anna E. Mattlage, Nicole M. Seier, Jonathan D. Todd, Brian G. Price, Sarah J. Kwapiszeski, Rakesh Vardey, Sandra A. Billinger

**Affiliations:** 1 University of Kansas Medical Center, Department of Physical Therapy and Rehabilitation Science, Kansas City, Kansas, United States of America; 2 University of Kansas Medical Center, Department of Physical Medicine and Rehabilitation, Kansas City, Kansas, United States of America; Kent State University, UNITED STATES

## Abstract

**Purpose:**

The purpose of the present study was to determine the reliability of the exercise response (predicted peak VO_2_) using the total body recumbent stepper (TBRS) submaximal exercise test in: 1) healthy adults 20–70 years of age and 2) adults participating in inpatient stroke rehabilitation. We hypothesized that the predicted peak VO_2_ (Visit 1) would have an excellent relationship (r > 0.80) to predicted peak VO_2_ (Visit 2). We also wanted to test whether the exercise response at Visit 1 and Visit 2 would be significantly different.

**Methods:**

Healthy adults were recruited from the Kansas City metro area. Stroke participants were recruited during their inpatient rehabilitation stay. Eligible participants completed 2 TBRS submaximal exercise tests between 24 hours and 5 days at similar times of day.

**Results:**

A total of 70 participants completed the study. Healthy adults (n = 50) were 36 M, 38.1 ± 10.1 years and stroke participants (n = 20) were 15 M, 62.5 ± 11.8 years of age. The exercise response was reliable for healthy adults (r = 0.980, p<0.01) and stroke participants (r = 0.987, p<0.01) between Visit 1 and Visit 2. Repeated Measures ANOVA showed a significant difference in predicted values between the two visits for healthy adults (47.2 ± 8.4 vs 47.7 ± 8.5 mL∙kg^-1^∙min^-1^; p = 0.04) but not for stroke participants (25.0 ± 9.9 vs 25.3 ± 11.4 mL∙kg^-1^∙min^-1^; p = 0.65).

**Conclusion:**

These results suggest that the exercise response is reliable using the TBRS submaximal exercise test in this cohort of healthy adults and stroke participants.

## Introduction

Submaximal exercise testing provides health care and exercise professionals the opportunity to predict VO_2_ peak, a measure of cardiorespiratory fitness. The American Heart Association published a scientific statement, *The Importance of Cardiorespiratory Fitness (VO*_*2*_ *max) in the United States*, which highlights that cardiorespiratory fitness is one of the strongest predictors of all- cause mortality and recognized as an important marker of function and overall cardiovascular health [1]. This method has been considered to be more feasible in a clinical setting especially since measuring metabolic gas exchange is often not available [2] and maximal exercise testing maybe poorly tolerated by clinical populations including stroke. Furthermore, the results of submaximal exercise testing can be reliable when standardized testing procedures are used [2, 3]. We previously developed a submaximal exercise test using a total body recumbent stepper (TBRS) and cross-validated the VO_2_ peak prediction equation in healthy adults [4] and in older adults [5].

The TBRS exercise device can be used in patient populations with balance and gait impairments such as stroke. This device has the ability to accommodate a variety of physical impairments [6–8] and has been suggested to be the preferred exercise modality for older adults [9]. Our early work demonstrated that peak exercise testing is valid using the modified TBRS-XT in chronic stroke [6] but exercise testing using metabolic gas is difficult during inpatient rehabilitation since most stroke units are not equipped to conduct this type of assessment. Submaximal exercise testing during inpatient stroke rehabilitation can be conducted to assess cardiorespiratory fitness in order to establish an individualized exercise program [10, 11].

In order to use the TBRS submaximal exercise test to assess cardiorespiratory fitness pre and post-exercise intervention, it is important to determine whether the exercise response (measured as predicted peak VO_2_) is reliable. The purpose of this study was to determine the reliability of the exercise response of the TBRS submaximal exercise test in healthy adults and stroke participants during inpatient rehabilitation. For our primary question, we hypothesized that the predicted peak VO_2_ (Visit 1) would have a significant and excellent relationship [12] (r > 0.80) to predicted peak VO_2_ (Visit 2). We also wanted to test whether the exercise response at Visit 1 and Visit 2 would be significantly different. We conducted this work in healthy adults first (experiment 1) and then in stroke participants during inpatient rehabilitation (experiment 2).

## Methods

### Participants

#### Experiment 1: Healthy adults

Individuals were recruited from the community using a flyer approved by the KU Medical Center human subjects committee. Once potential participants called the laboratory, they were screened for eligibility. In order to be included in the study individuals must: 1) be between 18 and 70 years of age, 2) have the ability to participate in maximal and submaximal exercise testing, and 3) have the ability to travel or have transportation to participate in the exercise testing sessions. Individuals were excluded from the study if they: 1) met criteria of high risk according to the American College of Sports Medicine (ACSM) [3] cardiac risk stratification categories, 2) were unable to physically perform the exercise tests using the treadmill and recumbent stepper, 3) demonstrated absolute indications for terminating exercise testing that follows the ACSM’s guidelines [3], 4) have a diagnosis of cardiovascular or coronary artery disease and/or respiratory disease; or 5) have a bone or joint problem that may be aggravated by maximal exercise testing. Those who met the inclusion criteria were invited to participate.

#### Experiment 2: Stroke participants

Physical therapists or the physician at KU Hospital identified stroke patients who met the inclusion criteria: 1) between 18 and 85 years of age, 2) participating in stroke rehabilitation at KU Hospital, and 3) ability to participate in submaximal exercise testing with clearance from physician. Exclusion criteria were: 1) inability to physically perform the exercise test using the recumbent stepper, 2) unstable cardiovasular or pulmonary signs/symptoms, 3) unstable hemodynamic response during therapy or at rest, 4) spasticity or presence of contracture in the affected limb that would not allow the arm or leg to bend and extend during the movement of the exercise device, and 5) demonstrate absolute indications for terminating exercise that follows the American College of Sports Medicine's guidelines [3].

Per KU hospital protocol, a clinician familiar with the patient or part of the healthcare team approaches the patient and gauges interest in learning more about the study. In this study, the physical therapist was the clinical contact between the patient and our laboratory team. The physical therapist would approach their patient to determine interest in the study. If the participant was interested, the stroke physician (R.V.) was notified and determined medical safety. The physician then signed the form indicating whether the patient was medically stable to participate in the TBRS submaximal exercise test. If eligible, a member of the research team approached the stroke patient to explain the study in full detail and obtain written informed consent.

The study procedures for this project were reviewed and approved by University of Kansas Medical Center Human Subjects Committee. Institutionally approved written informed consent was obtained from all participants prior to initiation of any study procedures.

### Procedures

All participants were instructed to avoid consuming food or drink, with the exception of water, two hours prior to each exercise test session. Additionally, participants were asked to avoid caffeine for 6 hours. Healthy participants were asked to avoid strenuous physical activity and maintain a similar lifestyle (e.g. sleep, exercise, diet) for 24 hours prior to the exercise test session. All participants were familiarized and practiced the reciprocal movement pattern and step rate of the TBRS submaximal exercise test on the recumbent stepper (NuStep, T5XR, NuStep, Ann Arbor, MI) 30–45 minutes prior to the initiation of the testing procedure. This occurred on Visit 1 only. After completion of the initial TBRS submaximal exercise test (Visit 1), the second session (Visit 2) was scheduled between 24 hours but no more than 5 days of the previous visit, with no more than one-hour difference in the time of day. Room temperature and humidity were recorded for each exercise session. (Table 1) Participants were instructed to avoid casual conversation during the TBRS submaximal exercise test unless related to the study procedure (perceived exertion scale). To ensure safety of our participants, we followed published guidelines for absolute and relative exercise test termination [3].

**Table 1:** Environment and physiologic measures.

Variable	Healthy Adults	p-value	Stroke Participants	p-value
Temperature (°C)				
Visit 1	23 ± 1	0.06	22 ± 2	0.89
Visit 2	23 ± 1		22 ± 2	
Humidity (%)				
Visit 1	31 ± 4	0.16	18 ± 14	0.21
Visit 2	30 ± 5		20 ± 15	
Resting HR (bpm)				
Visit 1	58 ± 10	0.05	80 ± 12	0.79
Visit 2	60 ± 10		79 ± 10	
Resting Systolic BP (mmHg)				
Visit 1	123 ± 14	0.28	132 ± 16	0.83
Visit 2	122 ± 13		132 ± 13	
Resting Diastolic BP (mmHg)				
Visit 1	72 ± 9	0.001	78 ± 6	0.67
Visit 2	68 ± 10		79 ± 10	
End Exercise HR (bpm)				
Visit 1	142 ± 15	<0.01	121 ± 14	0.85
Visit 2	141 ± 16		121 ± 14	
End Work (watts)				
Visit 1	153 ± 25	0.71	63 ± 22	0.33
Visit 2	153 ± 24		64 ± 25	
End RPE				
Visit 1	16 ± 2	0.56	16 ± 2	0.19
Visit 2	15 ± 2		16 ± 1	
End				
Visit 1	687 ± 83	0.67	337 ± 127	0.21
Visit 2	684 ± 81		350 ± 113	

°C = degrees Celsius; % = percent; HR = heart rate; bpm = beats per minute; BP = blood pressure; mmHg = millimeters of mercury, RPE = rate of perceived exertion

### Submaximal exercise testing

#### Experiment 1: Healthy adults

The consent, questionnaires and testing sessions were held at the University of Kansas Medical Center Research in Exercise and Cardiovascular Health (REACH) Laboratory. Height and weight were obtained at the beginning of each session. Each participant was fitted with a Polar heart rate (HR) monitor and chest strap for continuous HR monitoring throughout the TBRS submaximal exercise test. Resting blood pressure (BP) and heart rate (HR) were documented after sitting quietly for 15 minutes and prior to the start of the exercise test. Heart rate and BP were recorded again at the end of the exercise test.

The TBRS submaximal exercise test and VO_2_ prediction equation has been previously published [4]. Briefly, individuals were instructed to find a comfortable step rate between 90–100 steps per minute (spm) and then maintain that rate throughout the entirety of the test. The TBRS submaximal exercise test followed the Young Men’s Christian Association (YMCA) [13] protocol except participants began Stage 1 at 30 watts. The TBRS submaximal exercise test and prediction equation was valid in healthy adults [4] and in older adults with multiple chronic conditions [5]. The resistance was increased every three minutes for the remaining stages until: 1) the participant reached volitional fatigue or requested to stop, 2) 85% of age-predicted HR max (0.85(220-age)) was achieved; or 3) completed the TBRS submaximal exercise test [3]. Heart rate was recorded ten seconds before minute 2 and 3 of each stage. If these two heart rate measurements were within 5 beats per minute (bpm) of one another, the participant moved on to the next stage. If the two heart rate measurements were greater than 5 bpm from each other, the participant continued for an additional minute in that stage to ensure a steady state has been reached. After completion of the TBRS submaximal exercise test, participants stepped at 25 watts at a self-selected pace for 2 minutes then rested until HR reached near resting values. For experiment 1, the study personnel conducting the submaximal exercise test for Visit 2 was blinded to the results from a different rater at Visit 1.

#### Experiment 2: Stroke participants

Exercise testing was conducted at KU Hospital and only in the afternoon at least 1 hour after all therapy sessions were completed. For stroke participants, we used 80–90 steps per minute identical to our modified maximal exercise test step cadence for people after stroke [6]. If necessary, adaptive equipment (leg stabilizer and hand grip) was used for the stroke affected side. For experiment 2, study personnel (D.W., A.M., S.B.) conducted Visit 1 and Visit 2 procedures. To maintain blinding of the Visit 1 exercise response, Visit 1 data collection sheet was given to a non-tester (SK) and no data was entered until after completion of Visit 2. All other procedures for the TBRS submaximal exercise test were followed.

### Sample size justification

Studies of test-retest reliability have used sample sizes of five [14], ten [15] and up to 62 [16, 17]. For this study, we chose to use 50 healthy adults and 20 stroke participants. Future studies will have data from the TBRS submaximal exercise test to conduct a power analysis.

### Statistical analysis

The arithmetic mean and standard deviation were used for descriptive statistics. Pearson correlation coefficient was calculated to determine reliability of exercise response to the TBRS submaximal exercise test. For this study, we used criteria previously published by Portney and Watkins [12]: Pearson’s coefficient (r) = 0.00–0.25, little to no relationship; r = 0.25–0.50, fair relationship; r = 0.50–0.75, moderate to good relationship; and r >0.75, good to excellent relationship. To test the level of agreement between estimated peak VO_2_ for Visit 1 and Visit 2, Bland Altman plots were used. Repeated measures ANOVA was used to determine whether the predicted peak VO_2_ at Visit 1 was significantly different from the value obtained at Visit 2. In experiment 1, we assessed interrater reliability (ICC) using one-way random effects model. All analyses were conducted using SPSS statistical software (Version 20; SPSS, Inc, Chicago, IL) with the alpha level <0.05.

## Results

No adverse events were reported during the study. Differences in the environment and physiologic responses between Visit 1 and Visit 2 are presented in Table 1 for both groups.

### Experiment 1: Healthy adults

Fifty-six individuals were screened for participation. Of the 56 individuals, 6 were not eligible to participate for the following reasons: 1) determined to be high cardiac risk (n = 2) and 2) declined to enroll in the study during the prescreening phone call (n = 4). Fifty individuals met the inclusion criteria and were enrolled into the study. Demographic information is listed in Table 2. After completion of the initial TBRS submaximal exercise test (Visit 1), the second session (Visit 2) was scheduled between 24 hours but no more than 5 days of the previous visit for all participants and tests were completed with no more than one-hour difference in the time of day between Visit 1 and Visit 2 (S1 Appendix contains full data set).

**Table 2:** Participant demographics.

Characteristics n = 70	Healthy Adults (n = 50)	Stroke Participants (n = 20)
Sex: Male	36	15
Age (years)	38 ± 10	63 ± 12
Body Mass Index (BMI)	23.8 ± 3.4	28.6 ± 5.7
Race		
African American	3	4
Asian	0	1
Caucasian	46	15
Native American	1	0
Ethnicity		
Hispanic	0	1
Non-Hispanic	50	19
Risk Stratification for Cardiovascular Disease Risk		
Low	45	0
Moderate	5	0
High	0	20
Beta Blockers	0	2
Functional Independent Measure (Admission)		
Transfers	N/A	2 ± 2
Gait	N/A	1 ± 1

**Fig 1:**
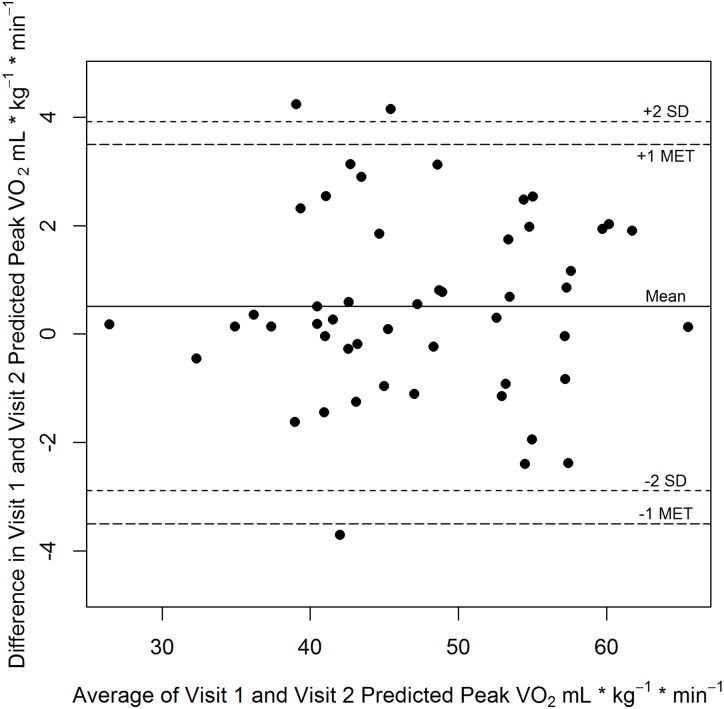
Bland-Altman plot of predicted peak VO_2_ values in healthy adults. Bland-Altman Plot of Predicted Peak VO_2_ for Visit 1 and Visit 2 in healthy adults.

The exercise response between Visit 1 and Visit 2 demonstrated a good to excellent relationship (r = 0.98, p<0.01). Results of the Bland Altman plot can be found in Fig 1. For the healthy adults, no systematic bias was observed and most differences in observations fell within 1 MET. Repeated Measures ANOVA showed a significant difference in predicted values between the two visits for healthy adults (47.2 ± 8.4 vs 47.7 ± 8.5 mL∙kg^-1^∙min^-1^; p = 0.04). The interrater reliability was good (ICC_(1,2)_ = 0.98, p <0.001; 95%CI = 0.96–0.99).

### Experiment 2: Stroke participants

Twenty-two people participating in in-patient stroke rehabilitation were enrolled into the study. Two individuals withdrew after consent. One person declined participation after consent and the other completed Visit 1 but declined to complete Visit 2. Data is reported on those who completed both visits (n = 20). Demographic information for those with stroke is presented in Table 2. The time between Visit 1 and Visit 2 was within 24–72 hours rather than 24 hours to 5 days as originally planned. The rationale for the decreased time between Visit 1and 2 was related to discharge planning from the hospital. To ensure that the stroke participants didn’t discharge prior to Visit 2, we chose to conduct the tests in a shorter timeframe. Despite intense rehabilitation schedules and medical tests, we conducted all exercise testing for the stroke participants with no more than one-hour difference in the time of day between Visit 1 and Visit 2 (See S2 Appendix).

**Fig 2:**
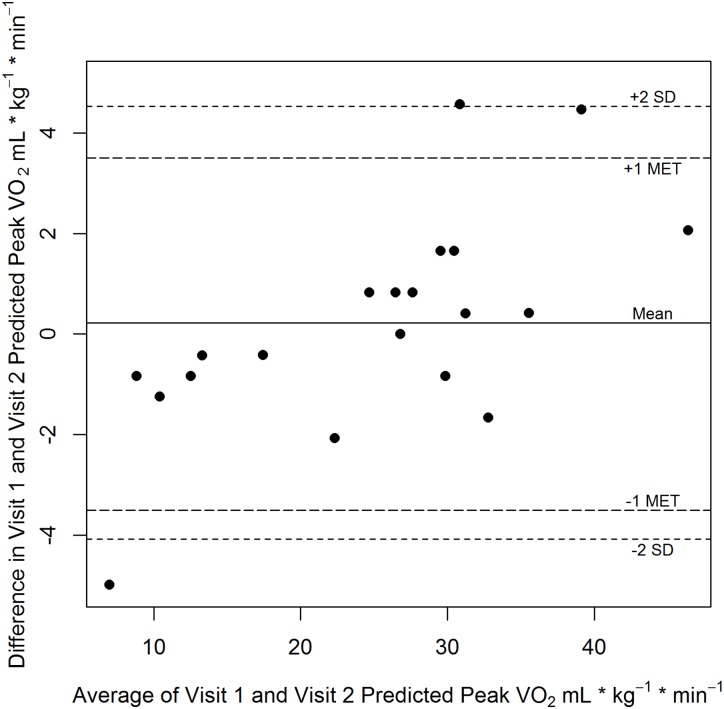
Bland-Altman plot of predicted peak VO_2_ values in stroke. Bland-Altman Plot of Predicted Peak VO_2_ for Visit 1 and Visit 2 in stroke.

The exercise response between Visit 1 and Visit 2 demonstrated an excellent relationship (r = 0.99, p<0.01). Results of the Bland Altman plot for the stroke participants can be found in Fig 2. For the stroke participants, our sample size may be insufficient to detect systematic bias but most differences in the observations fell within 1 MET. Repeated Measures ANOVA showed found no significant difference in predicted values between the two visits (25.0 ± 9.9 vs 25.3 ± 11.4 mL∙kg^-1^∙min^-1^; p = 0.65).

## Discussion

Our previous work has demonstrated that the TBRS submaximal exercise test is valid and accurately predicts peak VO_2_ in healthy adults [4], older adults [5] and in people post-stroke [18]. However, before the TBRS submaximal exercise test can be used to assess predicted peak VO_2_ pre and post intervention, it was necessary to determine whether predicted peak VO_2_ was reliable. We tested whether the predicted peak VO_2_ (Visit 1) would have an excellent relationship (r > 0.80) to predicted peak VO_2_ (Visit 2). The primary outcome of this study supports the reliability of the TBRS submaximal exercise test in healthy adults (20–70 years of age) and for stroke participants participating in rehabilitation. Despite the high levels of reliability observed in the healthy adults, the results suggested there was a statistically significant difference between the exercise response between Visit 1 and Visit 2. The mean difference was 0.5 mL∙kg^-1^∙min^-1^. Although statistically significant, the magnitude of the difference is unlikely to be clinically meaningful (as demonstrated in the Bland Altman plot) since changes of 1 metabolic equivalent (3.5 ml*kg^-1^*min^-1^) have been associated with meaningful outcomes [19, 20].

For the stroke participants, the mean difference (0.3 mL∙kg^-1^∙min^-1^) between Visit 1 and Visit 2 was not statistically different. The healthy adults had not used the recumbent stepper prior to the study but did practice prior to the initial testing session. All 20 stroke participants had experience using the exercise device as part of their usual care during rehabilitation and prior to initiation of our study but did practice the step rate prior to beginning the exercise test on Visit 1. There is also the possibility that temperature and humidity could affect performance. As observed in Table 1, neither temperature nor humidity was significantly different between the two visits but we did observe more variability in humidity especially in the inpatient hospital setting.

One of the strengths of the paper was in experiment 1 the second rater was blinded to the exercise response of the first rater at Visit 1. We found excellent test retest reliability with the healthy adults. In light of these strengths, several limitations should be noted. First, we were unable to maintain consistent schedules for different raters in experiment 2. This was primarily due to variation in the discharge date for the stroke participants and we only had 3 raters available who could work with the stroke participants during their inpatient hospital stay. Since there were only 3 raters and the same rater conducted the exercise test for Visit 1 and Visit 2, this may have reduced variability in the exercise response. Second, the stroke participants may have been comfortable with having the same person conduct both tests. In addition, the instructions by the rater may have been more consistent between Visit 1 and Visit 2 since it was the same rater. This is important to consider since the admission evaluation during inpatient rehabilitation may be performed by a different physical therapist than the one performing the discharge assessment. Therefore, future work should examine whether the exercise response of stroke participants is reliable when using different raters.

Our results suggest that the TBRS submaximal exercise test would be reliable to use with stroke patients during inpatient rehabilitation. We know that assessment for cardiorespiratory fitness following stroke is recommended [21] but not often performed by physical therapists due to balance, mobility and cardiac concerns [10, 22]. For the stroke participants, the average Functional Independence Measure (FIM) score for transfers was 2.53 ± 1.57 (range 1–7; with 1 = total assist and 7 = completely independent) while most participants required total assistance for walking (1.05 ± 1.19 (range 0–4; 0 = not assessed). Despite these lower levels of walking, we were able to conduct a submaximal exercise test. Our work supports the existing literature that people post-stroke with varying levels of function can safely transfer and use the recumbent stepper [6, 9, 23]. We do report a high level of test-retest reliability with the stroke participants but these results should be interpreted with caution. Our sample size is small and these individuals were selected by the individual physical therapists, which may result in a selection bias. The therapists would decide whether the stroke patient was an appropriate candidate based on the inclusion/exclusion criteria and their clinical judgment regarding the presence of aphasia and physical function.

We did not receive information regarding how many individuals were pre-screened prior to contact with the physical therapist to determine interest in the study. Once the stroke patient agreed to be contacted and the physician release for exercise participation was signed, then our team was notified and we approached the patient about the study and consent. For those participants who expressed interest and consented, 91% (20/22) completed the study. Future work will a larger sample of people post-stroke and determine whether the TBRS submaximal exercise test can be conducted on stroke participants upon admission to inpatient rehabilitation to assess baseline cardiopulmonary fitness. This is an important first step in identifying an exercise protocol in this clinical population that has a reliable exercise response across 2 visits on separate days.

## Conclusions

The TBRS submaximal exercise test can be used in healthy adults and those participating in inpatient stroke rehabilitation. Our results suggest that the exercise response was reliable for both groups participating in the TBRS submaximal exercise test but a practice session is recommended prior to the submaximal exercise test to minimize practice effects if multiple tests are conducted.

## Supporting information

S1 Appendix This includes data for the healthy adults. (SAV)

S2 Appendix This includes data for the stroke patients. (SAV)
